# Electroporation with Calcium or Bleomycin: First Application in an In Vivo Uveal Melanoma Patient-Derived Xenograft Model

**DOI:** 10.3390/ph17070905

**Published:** 2024-07-07

**Authors:** Ralitsa Anastasova, Miltiadis Fiorentzis, Hongtao Liu, Sami Dalbah, Nikolaos E. Bechrakis, Berthold Seitz, Utta Berchner-Pfannschmidt, Theodora Tsimpaki

**Affiliations:** 1 Department of Ophthalmology, University Hospital Essen, University of Duisburg-Essen, Hufeland Str. 55, 45147 Essen, Germany; ralitsa.anastasova@uk-essen.de (R.A.); hongtao.liu@uk-essen.de (H.L.); sami.dalbah@uk-essen.de (S.D.); nikolaos.bechrakis@uk-essen.de (N.E.B.); utta.berchner-pfannschmidt@uk-essen.de (U.B.-P.); theodora.tsimpaki@uk-essen.de (T.T.); 2 Department of Ophthalmology, Saarland University Medical Center, Kirrberger Str. 100, 66421 Homburg, Germany; berthold.seitz@uks.eu

**Keywords:** uveal melanoma, calcium electroporation, electrochemotherapy, bleomycin, patient-derived xenograft, chorioallantoic membrane assay

## Abstract

Uveal melanoma (UM) represents a rare tumor of the uveal tract and is associated with a poor prognosis due to the high risk of metastasis. Despite advances in the treatment of UM, the mortality rate remains high, dictating an urgent need for novel therapeutic strategies. The current study introduces the first in vivo analysis of the therapeutic potential of calcium electroporation (CaEP) compared with electrochemotherapy (ECT) with bleomycin in a patient-derived xenograft (PDX) model based on the chorioallantoic membrane (CAM) assay. The experiments were conducted as monotherapy with either 5 or 10 mM calcium chloride or 1 or 2.5 µg/mL bleomycin in combination with EP or EP alone. CaEP and ECT induced a similar reduction in proliferative activity, neovascularization, and melanocytic expansion. A dose-dependent effect of CaEP triggered a significant induction of necrosis, whereas ECT application of 1 µg/mL bleomycin resulted in a significantly increased apoptotic response compared with untreated tumor grafts. Our results outline the prospective use of CaEP and ECT with bleomycin as an adjuvant treatment of UM, facilitating adequate local tumor control and potentially an improvement in metastatic and overall survival rates.

## 1. Introduction

The common trait of cutaneous and non-cutaneous melanomas, found in the skin, eye, inner ear, and other internal organs, is the presence of melanocytes [1,2]. When stratified by anatomic region, cutaneous melanoma is the most prevalent type, followed by non-cutaneous uveal melanoma (UM), arising at ocular sites. UM represents over 80% of all ocular melanomas, whereas melanomas of conjunctival and orbital origins are less frequent [3]. The prevalent sites of melanoma in the uveal tract include the choroid (90%), the ciliary body (6%), or the iris (4%) [4]. The incidence of UM increases with age, and the diagnostic peak is reached between the sixth and seventh decades of life [5]. It is primarily found in Caucasians and is slightly more common in men [6,7,8,9]. In Europe, a north-to-south decreasing gradient, attributed to the protective role of ocular pigmentation in southern populations, accounts for an incidence rate ranging from two to eight cases per million per year [7,10]. UM has a high tendency to metastasize, most commonly in the liver (89%), lung (29%), and bones (17%), resulting in high mortality [11,12,13,14]. Irrespective of the selected treatment regimen, 50% of patients develop metastatic disease within 10 years of diagnosis [6,7,15]. Clinical parameters for high-risk patients include large tumor size, extraocular tumor extension, and advanced tumor staging [16]. Emerging as an important prognosis factor, the tumor cell type has been shown to correlate with the mortality rate, where spindle cell UM is linked to a better prognosis, mixed cell UM to an intermediate, and epithelioid cell UM may correspond with a poor outcome [17,18,19]. Genetic anomalies associated with chromosomes 1, 3, 6, and 8 play a pivotal role and indicate the survival rate in patients with UM. Monosomy 3, 1p loss, 6q loss, and 8q gain are predictors of poor prognosis [20,21,22]. Constitutive activation of the G-protein α subunit (Gα) signaling pathway involving the *GNAQ* and *GNA11* genes appears to have a distinct role in UM pathogenesis [23]. Furthermore, mutation of BRCA1-associated protein 1 (*BAP1*), splicing factor 3b subunit 1 (*SF3B1*), and eukaryotic translation initiation factor 1A X-linked (*EIF1AX*) predispose patients to develop UM [24,25].

The selection of a proper management for primary UM remains individualized to the needs of each patient and includes radiation delivery systems, such as brachytherapy and teletherapy radiation, or surgery in the form of transscleral resection, endoresection, and enucleation [26]. Despite initial local tumor control, the risk of distant recurrence in patients with UM remains high [27]. Metastatic UM is refractory to conventional chemotherapy and poses an urgent need for new adjuvant therapeutic strategies. Furthermore, the estimated lifetime risk of death and the survival rate are highly impacted following the diagnosis of metastatic disease [11]. In the constant pursuit of effective targeted therapeutic options, reliable in vivo models are required as an important tool for preclinical studies.

Electroporation (EP) refers to the increased permeability of the plasma membrane due to the formation of aqueous nanopores after the exposure of cells to short electric pulses with sufficient amplitude [28]. Irreversible EP, which leads to cell damage beyond repair and apoptosis, can be applied as a focal ablative approach to treat solid tumors, which is unsuitable for other treatment modalities [29]. In reversible EP, the permeabilization is transient, allowing the delivery of otherwise impermeant molecules into the cells, and the membrane reseals after a certain time, regaining homeostasis [30,31]. The use of reversible EP to temporarily increase membrane permeability and to enhance the transportation of chemotherapeutics, such as bleomycin or cisplatin, is defined as electrochemotherapy (ECT) [32]. ECT exhibits an improved effectiveness on tumor growth with lower drug doses than in conventional chemotherapy, which alone has minimal or no antitumor activity [33]. With an emphasis on its safety profile, availability, and low cost, calcium EP (CaEP) has been described as a novel treatment setting elucidating dramatic antitumor responses [34,35,36,37]. The homeostasis of calcium, as an essential messenger involved in numerous cellular processes, is severely affected when a supraphysiological concentration of calcium is introduced into the cell via electropermeabilization. The increased influx of calcium ions after EP causes acute and severe ATP depletion associated with increased cellular use of ATP, inhibition of ATP production in mitochondria, as well as loss of intracellular ATP through permeabilized plasma membranes [38,39]. ECT is a well-established local therapy for primary and metastatic tumors, including cutaneous melanoma, but the application of EP-based therapeutic modalities in UM lacks extensive research for its exploration as a promising treatment option.

The chorioallantoic membrane (CAM) assay provides a simple and versatile preclinical in vivo model to study tumor formation, angiogenesis, and metastasis. Emerging as a cost-effective and highly reproducible platform, the CAM assay allows the screening of potential therapeutic strategies and agents in a short time frame [40]. The CAM represents an easily accessible, rich, vascularized anatomical structure formed as an extraembryonic membrane by the partial fusing of the chick’s chorion and its allantois during embryonal development [41]. Due to the gradual process of gaining immunocompetence from a mostly immunodeficient chicken embryo before day 9 to a fully mature immune system on day 18, the CAM model enables the transplantation of xenografts with a low risk of rejection [42,43]. The highly dense vascular network of the CAM creates an ideal environment for rapid tumor growth 2–5 days after engraftment due to the ubiquitous supply of oxygen, nutrients, and growth factors, compared with 3–6 weeks in other animal models [44].

Although xenograft models derived from cancer cell lines partly mimic the in vivo structure of cancer, they lack the reproduction of tumor microenvironments and heterogeneity, thus having little genetic or functional resemblance to the tumors from which they originate [45]. Patient-derived xenograft (PDX) models are established by engrafting fresh tumors resected from individual patients into animals. The PDXs maintain intratumoral spatial and temporal heterogeneity and most accurately recapitulate the microscopic, genetic, and functional characteristics of their parental tumors, evolving as an effective experimental platform that precisely reflects drug efficacies in the clinical setting [46,47,48,49,50,51].

The need for novel, safe, and efficient treatment strategies that prevent further disease progression, increase survival potential, and improve quality of life is crucial for UM patients. This study aimed to evaluate CaEP as a potential novel UM ablation therapy for the first time in a UM PDX model based on the CAM assay [52]. To elucidate the cytotoxic action of CaEP, the comprehensive in vivo approach involved its comparison to ECT as an EP-based treatment modality combined with the chemotherapeutic drug bleomycin. Patient-derived tumor xenografts were treated with increasing concentrations of either calcium chloride (5 or 10 mM) or bleomycin (1 or 2.5 µg/mL) prior to EP to investigate the response and effectiveness of exposure to both applied agents in combination with high-voltage electric pulses. This methodology allowed the extensive analysis of the proposed therapy regimens, underlining the in vivo perspectives of CaEP and ECT in the treatment of UM tumors and emphasizing the advantages of PDX and the CAM assay itself as an appropriate link between in vitro and in vivo studies.

## 2. Results

### 2.1. Characterization of the Patient-Derived Xenografts

**Figure 1:**
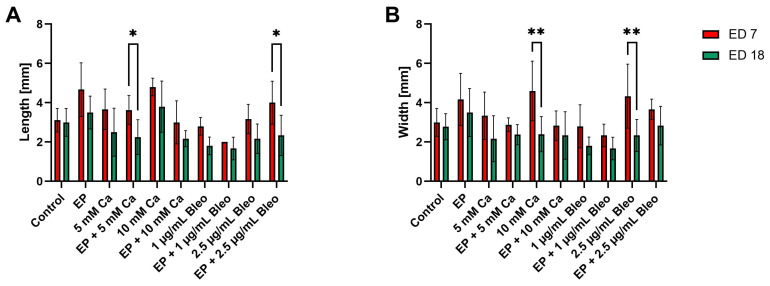
Characterization of the patient-derived xenografts among the various therapeutic modalities after measurements of the length and width before implantation on ED 7 and after excision on ED 18, n = 4–11 independent replicates per treatment condition. (**A**) EP in combination with either 5 mM calcium chloride or 2.5 µg/mL bleomycin revealed a significant decrease in length on ED 18 in contrast to ED 7. (**B**) The width of the tumor grafts on ED 18 was significantly reduced after application of either 10 mM calcium chloride or 2.5 µg/mL bleomycin compared with ED 7. Statistical analyses were performed using a two-way ANOVA and Sidak’s multiple comparison tests. Statistical significance levels are indicated with * *p* < 0.05, ** *p* < 0.01.

To compare the size of the engrafted tumor nodules among the different treatment settings in contrast to the untreated samples, their length and width were precisely measured before implantation on ED 7 and after excision on ED 18 (Figure 1A,B). In the control group, the implanted grafts demonstrated a mean length of 3.11 (±0.60 SD) mm and a mean width of 3.00 (±0.71 SD) mm before engraftment and 3.00 (±0.71 SD) mm and 2.78 (±0.67 SD) mm after dissection, which were not indicated as significant. The combinational treatment of 5 mM calcium chloride prior to EP had a statistically significant effect on the length on ED 18 compared with ED 7 (*p* = 0.0205). Similarly, a notable reduction in length was achieved after administration of 2.5 µg/mL bleomycin in combination with EP (*p* = 0.0125) (Figure 1A). The width of the tumor implants, when excised, was significantly decreased for both monotherapies, including injection of either 10 mM calcium chloride or 2.5 µg/mL bleomycin, compared with the implantation width (*p* = 0.0049 and *p* = 0.0051, respectively) (Figure 1B).

### 2.2. Histological Assessment of the Patient-Derived Xenografts

**Figure 2:**
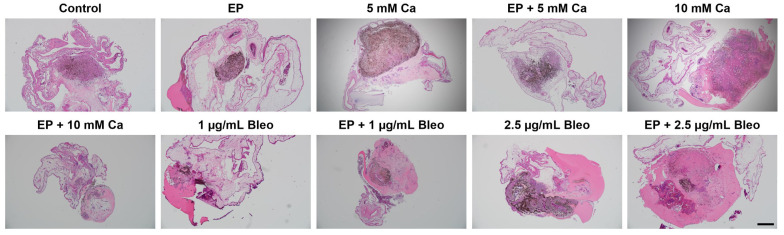
Morphological changes in response to the applied treatment modalities: representative images of haematoxylin and eosin staining of the patient-derived xenografts. Representative images illustrated morphological changes in response to the treatment settings. Successful integration of the tumor grafts with a regular cellular distribution was most prominent for the untreated tumor specimens. Detection of pigmented cells in the periphery was demonstrated mainly in the control and EP-only treated groups. Eccentrical tumor cell spreading recurred after application of calcium chloride monotherapies. In tumor samples treated with bleomycin alone, the peak of the tumor nodules showed a low cell density, and the main tumor body was predominantly positioned in the vicinity of CAM. Signs of tumoral dissociation, lower adhesion, and cellular loss in the tumor core were observed for the combined treatment conditions of either calcium chloride or bleomycin with EP. Scale bar represents 500 µm.

Hematoxylin and eosin staining served for the detailed visualization of phenotypical features of the tumor grafts (Figure 2). Hematoxylin stains nuclear structures in a dark blue-purple color, whereas eosin shows supportive structures surrounding cells in a pink-red color. The morphological evaluation revealed retainment of the pigmentation among all conditions. Tumoral integration into the CAM and intratumoral vascular growth were demonstrated for all samples, with a prominent folding of the surrounding tissue around untreated tumor nodules. Migration of pigmented cells in the proximal area of the CAM was observed predominantly in the control group, as well as after the application of only EP. A higher dispersion of tumor cells was observed for the calcium-chloride-only treatment. In tumor specimens solely treated with bleomycin, regions of uncolonized Matrigel were observed, whereas the peripheral cell distribution was predominantly detected in the proximal CAM. The histological assessment provided evidence of a possible induction of partial disorganization of the tumor structure with local depletion of cells intratumorally for both combined treatment options.

**Figure 3:**
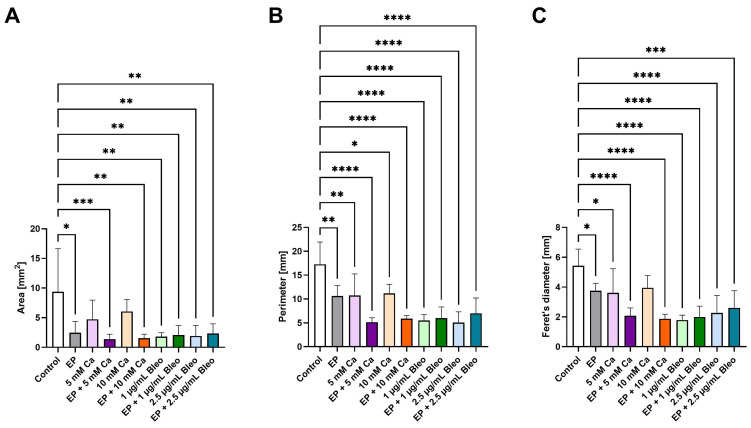
Comparison of size parameters of the patient-derived xenografts among the various therapeutic modalities after histological assessment. (**A**) The cross-sectional area was calculated as a measure of the tumor density. Exclusively for samples treated with either of the tested dosages of calcium chloride, no significant regress was stated compared with the control group. (**B**) To specify the outer tumoral boundary, the perimeter was analyzed. All treatments demonstrated a significant reduction in contrast to the untreated control. (**C**) The largest tumor diameter was presented through measurement of the Feret’s diameter. Besides administration of 10 mM calcium chloride, an apparent decline was observed across all remaining applied therapeutic settings in comparison to untreated tumor nodules. Statistical analyses were performed using a one-way ANOVA and Dunnet’s multiple comparison tests. Statistical significance levels are indicated with * *p* < 0.05, ** *p* < 0.01, *** *p* < 0.001, **** *p* < 0.0001.

The histological assessment was further used for quantitative analysis of dimensional changes. The cross-sectional area, perimeter, and Feret’s diameter were selected as tumor size parameters and were compared among the various therapeutic settings (Figure 3A–C). The area is a measure of tumor density, whereas the perimeter represents the distance around the tumor outline. As an additional variable, the Feret’s diameter indicates the maximum tumor spatial extent. Area measurements revealed a significant shrinkage for all conditions, including EP, as well as when bleomycin was applied alone compared with the untreated tumor grafts (3A). The highest statistically significant reduction in area was achieved after administration of 5 mM calcium chloride prior to EP (*p* = 0.0008). Across all tested treatment modalities, the perimeter was statistically decreased in contrast to the control group (Figure 3B). A less apparent effect on the perimeter was demonstrated after the application of 10 mM calcium chloride (*p* = 0.0388). Apart from the group treated solely with 10 mM calcium chloride, the Feret’s diameter was significantly reduced for all remaining treatment conditions in contrast to the untreated control (Figure 3C). In comparison to the other applied treatment setting, the monotherapy with EP and 5 mM calcium chloride led to a less noticeable difference in the estimated Feret’s diameter (*p* = 0.0486 and *p* = 0.0268, respectively).

### 2.3. Immunofluorescence Analysis of the Patient-Derived Xenografts

Immunofluorescence staining served the detailed analysis of implanted tumor graft viability and evaluation of the antitumoral effectiveness of the tested therapeutic modalities. Ki-67 and CD31 were selected as specific markers for the detection of proliferative capacity and neovascular action, respectively. Identification of tumor cells characteristic of melanoma was accomplished through an antibody cocktail indicated as melan-mix. The occurrence of cellular apoptosis and necrosis as a response to the applied treatments was assessed using caspase-3 and HMGB1 as suitable markers.

#### 2.3.1. Assessment of Proliferative Activity

The expression of Ki-67 is tightly correlated with cellular proliferation. Its distribution drastically changes during cell cycle progression, where it is highly expressed in cycling cells but absent in the resting cell cycle phase. This characteristic highlighted Ki-67 as a prominent proliferation marker for tumor cells. Immunofluorescence labeling with Ki-67 was used as a potent tool to assess tumor graft proliferative capacity.

**Figure 4:**
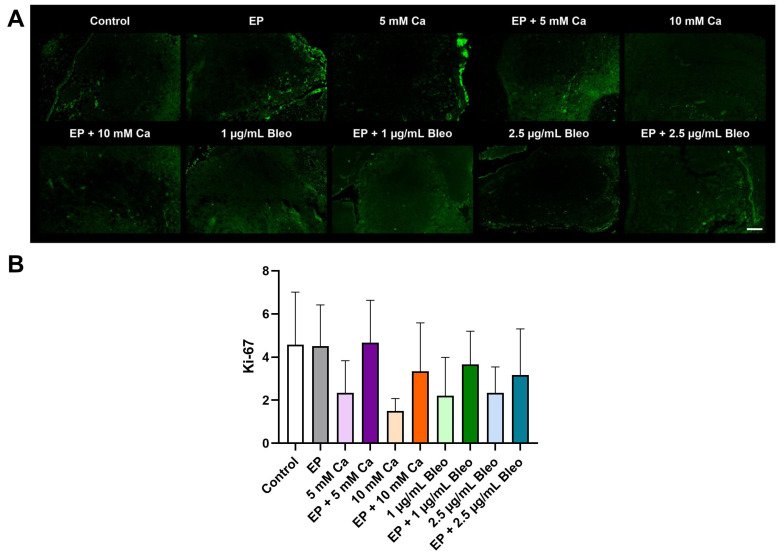
Analysis of Ki-67 expression for evaluation of proliferative activity of the patient-derived xenografts among the various therapeutic modalities after immunofluorescence staining; counts for positive cells per calculated area. (**A**) Representative fluorescence images demonstrated a weak positive signal across all groups with absence of signal in the central regions of the treated tumor grafts. Scale bar represents 100 µm. (**B**) Positive signals corresponded to positive cell counts. Quantification of the expression of Ki-67 revealed no significant change in proliferation activity after therapy compared with the control group. Statistical analyses were performed using a one-way ANOVA and Dunnet’s multiple comparison tests.

In all groups, including the untreated cohort, a low proliferative rate of the implanted tumor nodules was observed, potentially attributed to the slow proliferation of UM (Figure 4A). In contrast to the untreated tumor samples, for the other tested conditions, positive signals were mostly detected in the periphery of the tumor core. Quantitative evaluation of Ki-67 revealed no significant differences in cellular proliferation among the different conditions (Figure 4B). A regression of proliferation without statistical significance was observed in response to the application of calcium chloride or bleomycin monotherapies.

#### 2.3.2. Assessment of Neovascular Formations

The formation of a new vascular network to supply tumor tissue with substrates is fundamental for cell survival and growth. To assess neoangiogenesis in the implanted tumor grafts, the neovascular endothelial marker CD31 responding to emerging vessels was used.

**Figure 5:**
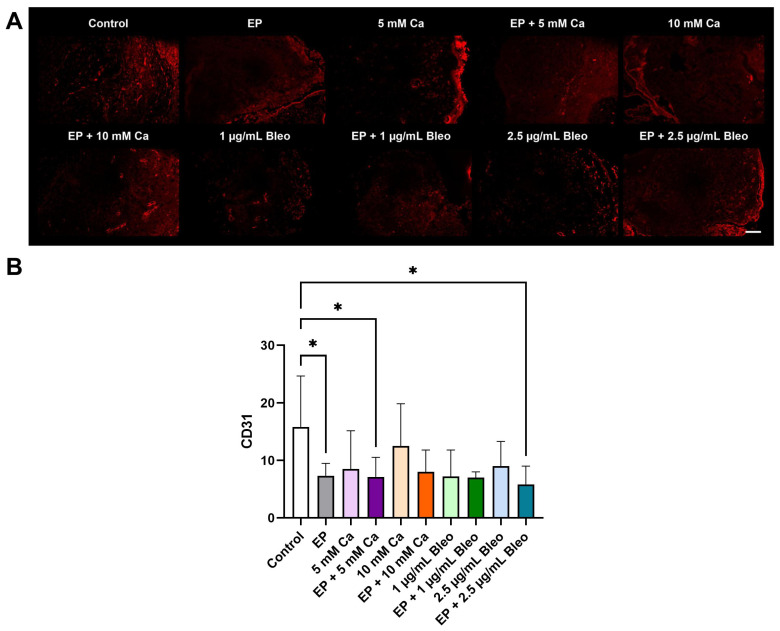
Analysis of CD31 expression for evaluation of neovascular formations in the patient-derived xenografts among the various therapeutic modalities after immunofluorescence staining; counts for positive cells per calculated area. (**A**) Representative fluorescence images demonstrated a strong positive signal in the control group localized both peritumorally and intratumorally. Weak positive staining distributed mostly across the CAM lining in the periphery of the tumor grafts was detected for the treated samples. Scale bar represents 100 µm. (**B**) Positive signals corresponded to positive cell counts. Quantification of the expression of CD31 revealed a significant decrease in CD31-positive cells in the groups treated with EP alone or in combination with either 5 mM calcium chloride or 2.5 µg/mL bleomycin compared with the untreated group. Statistical analyses were performed using a one-way ANOVA and Dunnet’s multiple comparison tests. Statistical significance levels are indicated with * *p* < 0.05.

Enhanced CD31 positive signals were detected in untreated samples with a homogenous distribution across the peritumoral and intratumoral areas (Figure 5A). In contrast, all treated tumor grafts, regardless of the therapy method, showed a declined expression of CD31, mostly located in the proximity of the CAM, and an absence of CD31 staining in tumor focal regions. Quantitative analysis revealed a significant decrease in CD31 positive counts after EP (*p* = 0.0453) (Figure 5B). Similarly, EP combined with either 5 mM calcium chloride or 2.5 µg/mL bleomycin induced a significant CD31 reduction (*p* = 0.0293 and *p* = 0.0123, respectively).

#### 2.3.3. Identification of Melanoma Cells

A triple antibody cocktail indicated as melan-mix, composed of HMB45 and two different clones of MART-1, was selected for the screening of melanoma cells in the engrafted tumor nodules. The coexpression of HMB45 and MART-1 facilitated a more accurate detection of melanomas due to the high sensitivity of HMB45 and the wide abundance of MART-1.

**Figure 6:**
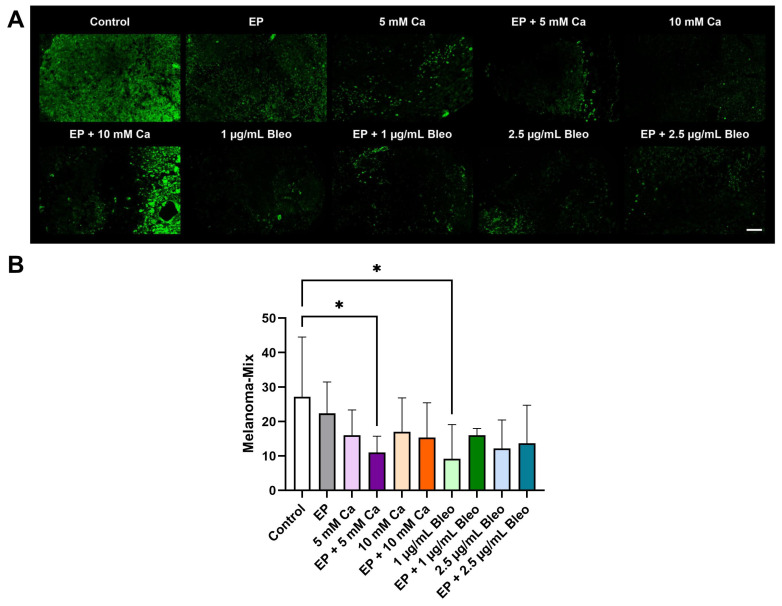
Analysis of melan-mix expression for identification of melanoma cells in the patient-derived xenografts among the various therapeutic modalities after immunofluorescence staining; counts for positive cells per calculated area. (**A**) Representative fluorescence images demonstrated a strong positive signal homogenously localized across the graft in the control group. Weak positive staining with characteristic absence from the focal tumor regions was detected for the treated samples. Scale bar represents 100 µm. (**B**) Positive signals corresponded to positive cell counts. Quantification of the expression of melan-mix revealed a significant decrease in melan-mix-positive cells in the groups treated with EP in combination with 5 mM calcium chloride or monotherapy with 2.5 µg/mL bleomycin compared with the untreated tumor fragments. Statistical analyses were performed using a one-way ANOVA and Dunnet’s multiple comparison tests. Statistical significance levels are indicated with * *p* < 0.05.

A prominent reduction in melan-mix-positive signals was predominantly detected in the tumor core following the various treatment settings, whereas a homogenous distribution of intense positive melan-mix expression was observed in the untreated cohort (Figure 6A). A significant decline in melanoma cell counts was noted for the group tested for the combinational therapeutic effect of EP and 5 mM calcium chloride (*p* = 0.0426) (Figure 6B). The application of 2.5 µg/mL bleomycin also induced an evident reduction in expression density (*p* = 0.0367).

#### 2.3.4. Assessment of Apoptotic and Necrotic Induction

Caspase-3 is a protease that coordinates the destruction of cellular structures in an apoptotic scenario. As an inactive precursor, caspase-3 has no executional functionality. During apoptotic signaling events, caspase-3 is cleaved, emerging as a suitable option for the evaluation of apoptotic induction in response to antitumoral therapeutic modalities. HMGB1 is a highly abundant nuclear protein that is passively released from damaged necrotic cells. HMGB1 served as a unique marker mediating the necrotic rate after application of the various treatment settings.

**Figure 7:**
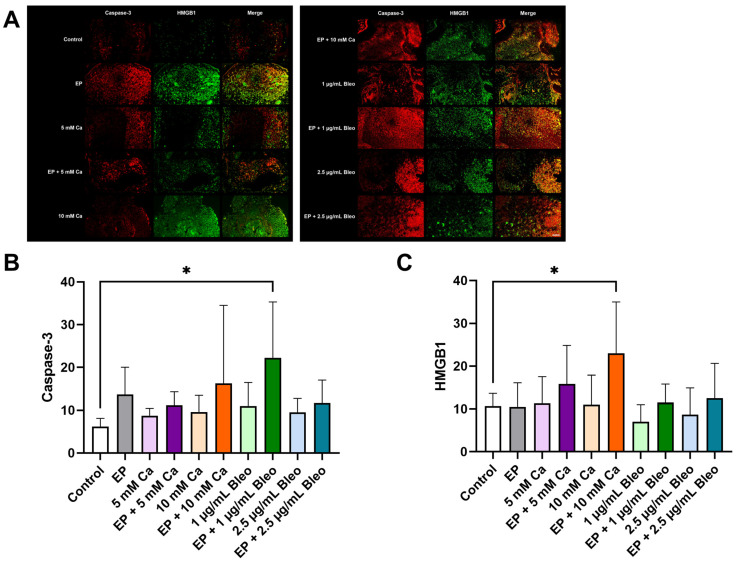
Analysis of caspase-3 and HMGB1 expression for evaluation of apoptotic and necrotic induction in the patient-derived xenografts among the various therapeutic modalities after immunofluorescence staining; counts for positive cells per calculated area. (**A**) Representative fluorescence images demonstrated an enhanced positive signal compared with the control group, particularly when EP was applied. Scale bar represents 100 µm. (**B**) Quantification of the expression of caspase-3 revealed a prominent induction of apoptotic signaling after administration of 1 µg/mL bleomycin prior to EP. (**C**) Positive signals corresponded to positive cell counts. A significant increase in the necrotic rate was achieved for tumor grafts treated with EP in combination with 10 mM calcium chloride compared with the untreated tumor fragments. Statistical analyses were performed using a one-way ANOVA and Dunnet’s multiple comparison tests. Statistical significance levels are indicated with * *p* < 0.05.

Compared with the untreated tumor grafts, a prominent increase in fluorescence signal was achieved, especially in the settings containing EP (Figure 7A). A visual appearance of multiple cell layers was observed in the tested conditions, including EP alone, administration of 5 mM calcium chloride alone, or combinational therapy of 1 µg/mL bleomycin prior to EP. Similar expression levels and patterns were noted for both markers. A significant increase in the apoptotic response was demonstrated in the group treated with EP after the application of 1 µg/mL bleomycin (*p* = 0.0241) (Figure 7B). Injection of 10 mM calcium chloride before EP expanded the necrotic rate (*p* = 0.0337) (Figure 7C).

## 3. Discussion

As the comprehensive exploration of the cellular and molecular pathomechanisms of UM progresses, the therapeutic regimen has gradually shifted toward globe-preserving choices, such as local surgical excisions combined with radiotherapy. Despite recent advances in diagnosis and management, a standardized, efficient therapeutic algorithm has not yet been established for the adequate control of metastatic disease, leading to dismal outcomes in approximately 50% of these patients [53,54]. Furthermore, there is scope for improvement in early diagnosis and prompt treatment initiation before the detection of metastatic lesions since the size of the primary tumor and adequate local tumor control are critical factors associated with survival. Taking the high metastatic risk and poor prognosis into consideration, the development of new in vivo PDX models for the improved understanding of UM tumor biology and dissemination, as well as of novel adjuvant treatment protocols, remains crucial. The current study is the first investigation of CaEP and ECT with bleomycin in patient-derived UM samples in an in vivo CAM model.

An optimal animal model that closely reflects UM pathogenesis and cellular behavior, mimicking the processes of tumor initiation, growth, metastasis, and the response to treatment as observed in patients, is an essential component of UM translational studies. Many attempts have been made to develop innovative and clinically relevant in vitro and in vivo preclinical models, and each one has distinctive strengths and weaknesses. Spontaneous-occurring ocular melanomas have been reported in the eyes of various animals, such as dogs and cats [55,56,57]. Although these tumors grow under natural conditions, their shortcomings associated with limited numbers of animals, unpredictable incidence rates, and inconsistent metastatic patterns restrict their use as a model for experimental UM research. Transgenic models provide a great option for monitoring the early stages of tumor growth but have variable metastatic profiles and shortened lifespans and require long generation times [58,59]. In induced animal models, tumors are artificially formed by the application of chemical agents, radiation, viruses, cells, or tissues, offering easy manipulation, improved reproducibility, and possible standardization [60]. A prominent limitation of the latter model includes the need for immunosuppression to avoid undesired immune responses. As an attractive alternative, the CAM assay can be advantageous due to its immunodeficient properties since immune cells become fully mature after hatching of the chicken embryos. Thus, transplantation procedures during the earlier incubation period frequently lead to a successful implantation and a faster growth rate than in other conventional animal models [61]. The use of the CAM assay is minimally invasive to the chicken embryo. Due to the lack of pain perception, the CAM is regarded as an ethical model in agreement with the 3R principle (reduction, refinement, and replacement), which does not require permission or approval from ethics committees [62]. The CAM system provides a unique biological microenvironment and a rich vascular network appropriate for cancer cells. A construct on the CAM is easily visualized and accessed. Different administration routes can be performed, including intravenous or topical. Beyond its technical and practical simplicity, as well as cost-effectiveness, the highly vascularized CAM enables the efficient engraftment of cells and tissue samples from various species, fast tumorigenesis, and the observation of metastasis and angiogenesis. The CAM assay serves as a powerful tool for the rapid assessment of the effectiveness of novel drug candidates for precision cancer therapy. Despite the short investigation period as a general limitation, it represents an intermediate step, linking in vitro and in vivo studies, and reducing the failure rate of anti-cancer drugs in clinical trials [63].

A standardized procedure for the CAM assay, proposed by several studies, was followed, which facilitated reproducibility of the experimental conditions. Sokolenko et al. optimized the CAM assay for the implantation of three-dimensional (3D) UM spheroids, improving embryo viability from 20% to >80% [64]. Furthermore, the modified protocol enabled the refinement of spheroid stability, vascularization, and volume. In another study, the CAM was used to investigate the effect of hypoxia preconditioning of UM cells on tumor growth and metastasis [65]. Our group postulated the CAM assay as a suitable platform to test the application and efficacy of CaEP, as well as ECT with bleomycin, on UM cell lines, emphasizing its suitability for the potential refinement of preclinical investigations and as a reliable intermediate step between in vitro cell and in vivo animal experiments in UM research [36,66]. For the establishment of the UM PDX model based on the CAM assay, various implantation techniques were analyzed, demonstrating high-efficiency engraftment that allows a precise, quantitative assessment [52]. The tumor grafts acquire the blood supply by the formation of new blood vessels from the host microvasculature. A progressive integration and successful implantation of the grafts were established when a vascular star was formed around the tumor approximately 1 week after engraftment.

Preclinical cancer research traditionally relies on in vitro platforms using monolayers of cell culture or in vivo animal models. Two-dimensional (2D) cell culture systems are routinely employed for initial studies due to easy handling and standardized methodologies but are far too simplistic to recapitulate the complexity of a tumor microenvironment [67,68]. However, growing 3D cell constructs, such as spheroids, mimics more closely the physical and biochemical features of solid tumor masses [69]. Nevertheless, 3D cell cultures cannot fully represent reliable preclinical models due to their low reproducibility, oversimplified tumor behavior and treatment response, as well as the lack of important factors, such as the presence of stromal and vascular components [70]. Common methods for tumor transplantation on the CAM include the deposition of a cell suspension, a spheroid, or excised tumor samples. The implantation of tumor tissues on the CAM allows the investigation of tumor development, angiogenesis, and treatment responses. Such PDX models include the engraftment of freshly harvested tumor specimens onto the CAM, preserving the microenvironment and metastatic potency [71]. In addition, PDX models enable a detailed analysis of molecular and cellular responses to treatment due to the retention of heterogeneity and pathophysiological features of the patient [72]. Within these advantageous characteristics lies the novelty of the present study since it analyzed the first results after the application of two EP-based treatment modalities in UM samples from patients.

ECT comprises the administration of chemotherapeutic agents in combination with electric pulses and has been widely used for the adjuvant treatment of various tumor entities [73,74,75,76,77]. As an alternative approach to traditional non-surgical therapeutic options for adequate local tumor control, ECT increases membrane permeability, enhancing the uptake and accumulation of drugs, otherwise not permeable, resulting in a dramatic rise in cellular cytotoxicity [78]. Regarding its application in ocular oncology, ECT has been described in preclinical in vitro and in vivo models, as well as in a few preliminary clinical studies. Fiorentzis et al. investigated the effect of bleomycin and cisplatin prior to EP in four UM cell lines, Mel 270, UM92.1, OMM-1, and OMM-2.5, delivering a greater response and lower resistance to ECT with bleomycin compared with cisplatin [79]. In another study, the same group used 3D spheroids from primary and metastatic UM cell lines, MP 46, UM92.1, Mel 260, MM28, and OMM-1, in vitro as well as after implantation in the CAM assay, showing a significant reduction in tumor size and tumor cell viability after therapy with bleomycin combined with EP in comparison to antineoplastic agent alone [66]. Furthermore, encouraging data from the treatment of three patients with basal cell carcinoma of the periocular region reported a complete resolution with minimal scarring and maintenance of the eye function [80]. The use of ECT as an adjuvant therapy for ocular melanoma has been demonstrated in a dog, leading to a complete tumor remission [81].

CaEP represents a novel antitumor treatment modality that offers several advantages, including low cost, long durability, and the absence of severe adverse effects. In 2012, Frandsen et al. proposed a preclinical proof of concept for a direct intratumoral calcium chloride injection followed by EP, resulting in a significant loss of tumor volume post-treatment [34]. Five years later, the first clinical trial was executed for patients with cutaneous metastases from breast cancer and malignant melanoma, outlining the safety and efficiency of CaEP [35]. Despite recent advances in the preclinical and clinical investigations of CaEP in different tumor entities, its potential has been limitedly covered in ocular tumors [82,83,84,85]. A dose-dependent reduction in cell viability and proliferative capacity has been observed in our previous study after CaEP in 2D UM cell lines and 3D UM cell spheroids derived from UM92.1, Mel270, UPMD2, and UPMM3 [37]. Similarly, Tsimpaki et al. emphasized the therapeutic prospective of CaEP in comparison to ECT with bleomycin using two UM cell lines, UPMD2 and UPMM3, in the CAM model, indicating its efficacy for the UM therapy application, in contrast to monotherapeutic approaches [36].

The concentrations of the applied drug agents were selected based on results from our previously published studies with ECT and CaEP. UM cell lines were shown to be more resistant to EP combined with cisplatin than with 1 µg/mL bleomycin, which led to the greatest decline in cell viability [79]. A similar cytotoxic effect was reached with both 5 and 10 mM calcium chloride prior to EP on the CAM, whereas a dose dependency was observed for ECT with bleomycin between 1 and 2.5 µg/mL [36]. A customized electrode designed for the treatment of 3D spheroids enabled the application of electric pulses without manual disturbance of the tumor masses through procedural manipulation [86].

In the current study, the intense pigmentation of all implanted patient-derived grafts enabled a better evaluation of the experimental implementation, including sample integration into the host vasculature, as well as precise drug administration. For all treatment conditions, especially when combined with EP, a positive response regarding the tumor size and configuration arose, which was additionally verified after histological assessment. Apart from Ki-67, all immunofluorescence markers used for the evaluation of cell viability and cytotoxicity after the applied, tested conditions generated significant results, predominantly for treatment settings combined with EP. A low proliferation rate was detected in both treated and untreated tumor grafts, which was speculatively attributed to the poor proliferative behavior of UM cells. In the study of Mooy et al., the Ki-67 score of non-irradiated UM varied between 0.16 to 3.08%, supporting the hypothesis of the low proliferative capacity in our study [87]. The detection of significant differences among such low numbers of positive cells becomes unattainable and may undermine the validity of a significant result if present. In addition, Char et al. investigated 30 patients with choroidal melanoma and suggested that small, thin tumors revealed a lower proliferation, whereas thicker tumors enlarged more rapidly [88]. The specimens in this study predominantly included thinner, medium-sized tumors, whereas two showed a thicker extraocular growth with less prominent intraocular components, and one displayed an inhomogeneous, partly amelanotic configuration. Therefore, a further caveat to stress in interpreting the low proliferative rate is whether the analyzed tumor samples were representative of typical UM size characteristics.

All treated groups exhibited a reduced ability for neovascular formations, according to the immunofluorescence analysis, in comparison to the control cohort, with a more prominent effect for EP alone or combined with either 5 mM calcium chloride or 2.5 µg/mL bleomycin. Lv et al. studied the effect of EP on both normal and tumor blood vessels, predominantly focusing on endothelial cells [89]. Tumor cells were considered more susceptible to EP, whereas normal blood vessels remained intact, attributed to the presence of vascular smooth muscle cells only in normal blood vessels and their protective role over endothelial cells. Another study discussed that CaEP affected normal and tumor blood vessels to the same degree in an in vivo setting [90]. The effect was more prominent on small blood vessels, leading to the inhibited migration and ability of endothelial cells to form a vascular network. Sersa et al. described a multipotent effect of ECT with bleomycin on vasculature, reporting an increased exposure of endothelial cells lining the tumor blood vessels to approximately 40% higher electric field in comparison to surrounding tissue, enhancing drug uptake into the cytosol [91]. Furthermore, the authors provided evidence for a dual vascular disruption following ECT, an immediate, short-lasting tumor blood flow reduction due to endothelial cell swelling, and an additional delayed vascular action due to the exposure to bleomycin causing endothelial cell apoptosis, inducing tumor necrosis and growth regression. The detection of significant differences not only after EP in conjunction with 5 mM calcium chloride and 2.5 µg/mL bleomycin but in all combined treatment concentrations may require a larger sample size per group.

Regarding the effect on the eradication of melan-mix-expressing cells, a great reduction in positive signal was achieved in all treated groups, most prominently after the administration of 5 mM calcium chloride prior to EP, as well as after monotherapy with 1 µg/mL bleomycin. In plant-based models, the process of browning and formation of melanin has been associated with the release of phenoloxidase after damage to the cell membrane, such as in EP [92]. In our study, an increased accumulation of pigmentation was noted intratumorally as well as in the surrounding CAM area, especially in EP-based conditions. This finding may be accredited to the release of melanin in the extracellular space due to the aforementioned cell membrane damage or to concurrent blood vessel disruption with subsequent extracellular hemosiderin deposition following EP.

A visible increase in both apoptotic and necrotic rates was induced in all groups treated with both combinational EP approaches, whereas the effect in monotherapy groups appeared less prominent. Although similar apoptotic and necrotic responses were observed in all tested treatment conditions, there was a tendency of a higher necrotic signal around the tumor edges, which were closer to the electrodes, where electric fields reached higher amplitudes and cells potentially underwent more extensive ablative damage [93]. Several studies supported that cell death after CaEP is not induced by apoptotic pathways [34,38,39,90]. On the contrary, the underlying cellular mechanisms regarding its cytotoxic effects are associated with intracellular ATP depletion and induction of necrosis, verifying the significant increase in the necrosis marker for the group treated with 10 mM calcium chloride and EP. Activation of apoptotic mechanisms linked to the accumulation of bleomycin following EP was demonstrated with a significant increase for 1 µg/mL of bleomycin. Thus, our results, in accordance with previous findings, suggested that CaEP was accompanied by a higher necrotic rate, whereas ECT with bleomycin led to a greater apoptotic reaction.

Our in vivo model incorporates the advantages of a PDX model and contributes to the development of personalized medicine. Despite its short lifespan, it offers the refinement of preclinical experimentation and the preselection of treatment settings, enabling the establishment of individualized therapeutic algorithms. EP-based treatment modalities constitute an alternative adjuvant treatment option for patients with UM, aiming for a more adequate local tumor control and, therefore, a reduction in metastatic and enucleation rates. In the current study, both CaEP and ECT with bleomycin displayed a similar efficacy regarding proliferation, alterations of the vascular tumor supply, and induced cell death, with varying sensitivity concerning the different applied agent concentrations. Furthermore, both approaches led to considerable shrinkage and alterations of the cellular morphology of the tumor samples. Thus, both CaEP and ECT with bleomycin represent possible alternative tools in the portfolio of the ophthalmic surgeon, facilitating an optimal selection of treatment. Further animal studies, as well as the conceptualization of electrodes suitable for the human eye, are required to allow the application of CaEP and ECT with bleomycin as therapeutic strategies for UM in a clinical setting.

## 4. Materials and Methods

### 4.1. Chicken Chorioallantoic Membrane Assay

Fertilized brown chicken eggs were purchased from Lohmann Deutschland (Ankum, Germany). Upon delivery, the eggs were cleaned with 50% ethanol to avoid contamination. This time point was assigned as day zero of embryonic development (ED 0). Eggs were stored in an upright position with the pointed side facing downward in an incubator (Bruja 3000 digital, Siepmann, Herdecke, Germany) at a temperature of 37.7 °C, a humidity of 60%, and a once-per-hour scheduled rotation. On ED 5, the rotation was discontinued, and 4–6 mL of albumin was removed to drop the CAM using a sterile syringe (BD Discardit II syringe, Becton, Dickinson and Company, Franklin Lakes, NJ, USA) and a sterile safety butterfly needle (Safety-Multifly needle, Sarstedt, Nümbrecht, Germany). The puncture side was covered with surgical tape (Micropore surgical tape, 3M, Saint Paul, MN, USA). On ED 6, a small window was cut in the shell under aseptic conditions, exposing the CAM. Sterile parafilm (Bemis Company Inc., Neenah, WI, USA) was placed over the hole, and the eggs were further incubated without rotation until implantation on ED 7.

### 4.2. Patient-Derived Xenografts

Fragments of freshly resected tumor specimens were obtained from ten UM patients after having undergone enucleation. The uveal melanoma samples were obtained from 7 men and 3 women who underwent an enucleation following the initial diagnosis. The mean age of the donors was 73.4 (±11.96) years. The histopathological analyses revealed UM of epithelioid cell morphology in three out of four monosomy 3 patients, whereas the rest of the patients showed a spindle cell shape and disomy 3. One patient did not sign the consent form, so no genetic data were available. On ED 7, the collected tumor samples were immediately incubated in medium (RPMI 1640 medium, Gibco, Thermo Fisher Scientific, Waltham, MA, USA) and stored on ice until implantation. Prior to engraftment, the CAM area was gently lacerated at a prominent vascular bifurcation using a spoon-shaped curette. Subsequently, 30 µL of Matrigel (Matrigel matrix, Corning, NY, USA) was pipetted directly on the lacerated region. The patient-derived grafts were implanted with forceps on the Matrigel droplet. Sterile parafilm was used to reseal the windows, and incubation continued until treatment on ED 14. A total of 126 eggs were implanted with one UM patient-derived xenograft. Tumor growth, CAM integration, and embryo viability were assessed by daily visual inspection.

Each treatment setting was tested on ED 14 in four to eleven independent biological replicates. Different concentrations of application solutions were prepared with either 5 or 10 mM calcium chloride (calcium chloride dihydrate Biochemica, AppliChem GmbH, Darmstadt, Germany) or 1 or 2.5 µg/mL bleomycin (bleomycin sulfate from *Streptomyces verticillus*, Sigma-Aldrich, St. Louis, MO, USA). Monotherapies included injection of 50 µL of either calcium chloride or bleomycin alone or EP alone using a customized voltage pulse generator (Cliniporator, IGEA S.p.A., Carpi, Italy). The two parallel aluminum electrodes were positioned 4 mm apart. Each electrode had a diameter of 1 mm and a length of 8 mm. When combined in the according groups, calcium chloride or bleomycin was administrated intratumorally prior to EP. The electrodes were placed on the CAM around the corresponding tumor graft. Eight rectangular pulses with a pulse duration of 100 µs, a 5 Hz repetition frequency, and a 1000 V/cm pulse strength were applied. Untreated tumor samples were used as controls. The specific electroporation setting was selected after previous investigations of our group in vitro.

**Figure 8:**
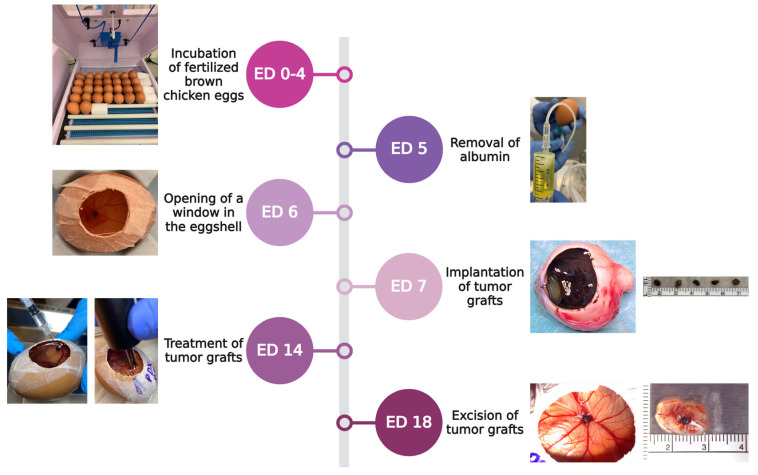
Experimental timeline according to the embryonic development of the fertilized chicken eggs starting with incubation upon arrival between ED 0 and ED 4, removal of albumin on ED 5, cutting a window in the shell on ED 6, engraftment of tumor samples harvested after enucleation on ED 7, followed by treating the tumor grafts on ED 14 via injection of calcium chloride or bleomycin alone, in combination with EP or EP alone. On ED 18, the tumors were excised, and the embryos were sacrificed.

On ED 18, a total of 71 implanted tumor grafts were excised, and photographic documentation was performed with a digital microscope camera (Leica IC80 HD, Leica Microsystems, Wetzlar, Germany) attached to a stereomicroscope (Leica M80, Leica Microsystems, Wetzlar, Germany). The embryos were sacrificed by decapitation. The lower CAM was examined for signs of secondary tumor sides and pigmentation (Figure 8).

### 4.3. Assessment Assays

#### 4.3.1. Characterization of the Patient-Derived Xenografts

The length and width of the tumor grafts were accurately measured with a surgical ruler before engraftment on ED 7 and after excision on ED 18. Photographic documentation was conducted.

#### 4.3.2. Preparation of Paraffin Sections

Following excision, the tumor samples were placed in embedding cassettes, fixated in 4% formaldehyde (ROTI Histofix, Carl Roth, Karlsruhe, Germany) overnight, and stored in 1% phosphate-buffered saline (Sigma-Aldrich, St. Louis, MO, USA) until further use. Specimens were immersed in graded series of alcohols and xylene for dehydration, embedded in paraffin, and sectioned at a thickness of 5 µm. The freshly cut paraffin sections were placed on microscope slides and dried overnight at room temperature. Prior to staining, the slides were deparaffinized.

#### 4.3.3. Histology

To demonstrate histological characteristics, hematoxylin and eosin staining was performed. Morphological and structural changes, integration in the surrounding CAM tissue, cell growth and migration, as well as size parameters were assessed after visualization using the brightfield settings of a fluorescence microscope (Olympus BX51, Olympus Corporation, Tokyo, Japan). The open-source image processing software ImageJ 1.54g (National Institutes of Health (NIH), Bethesda, MD, USA) was used to analyze the histological images. The size parameters of area, perimeter, and Feret’s diameter were estimated after the application of the various treatment modalities.

#### 4.3.4. Immunofluorescence

Immunofluorescence staining was conducted to evaluate cell viability and cytotoxicity using characteristic markers. Deparaffinized sections underwent antigen retrieval and blocking. Samples were incubated with primary antibody solutions at 4 ◦C overnight, followed by washing steps and incubation with secondary antibody solutions at room temperature for 30 min protected from light. For the assessment of cell proliferation, Ki-67 (1:150, Cell Signaling Technologies, Danvers, MA, USA) was used. Recognition of neovascularization was obtained by CD31 (1:50, Invitrogen, Waltham, MA, USA). Melanoma cells were identified using an antibody cocktail (1:100, Abcam, Cambridge, UK) referred to as melan-mix, comprised of HMB45 and two different clones of MART-1 (M2-7C10 and M2-9E3). Caspase-3 (1:400, Cell Signaling Technologies, Danvers, MA, USA) served for the visualization of apoptotic cell formations, and HMGB1 (1:400, Abcam, Cambridge, UK) for the detection of necrosis. Goat anti-mouse secondary antibody Alexa Fluor 488 conjugate and goat anti-rabbit secondary antibody Alexa Fluor 594 conjugate (1:400, Invitrogen, Waltham, MA, USA) were introduced. DAPI (1:1000, Carl Roth, Karlsruhe, Germany) was used for counterstaining. Washed slides were covered with fluorescence mounting medium (ProLong gold antifade mountant, Invitrogen, Waltham, MA, USA) and microscope cover glasses. Samples were visualized with a fluorescence microscope. The expression of the applied markers was quantified using ImageJ software. Positive signals corresponded to positive cell counts. Each image was assessed with a standardized count shape in three different areas of the immunofluorescence staining, and the mean value was calculated.

### 4.4. Statistical Analysis

Statistical analyses of the experimental data were performed using GraphPad Prism 9.5.1 software (GraphPad Software, San Diego, CA, USA). Statistical significances were assessed by one-way or two-way ANOVA tests. A value of *p* < 0.05 was considered statistically significant. Significance levels were indicated as * *p* ≤ 0.05, ** *p* ≤ 0.01, *** *p* ≤ 0.005, **** *p* ≤ 0.001.

### 4.5. Ethical Approval

The ethics committee at the Medical Faculty of University of Duisburg-Essen approved the study with the number 21-9959-BO. The study was conducted according to the Declaration of Helsinki and relevant local guidelines and regulations.

## 5. Conclusions

This is the first study using UM specimens from patients in vivo for the preclinical evaluation of the effectiveness of CaEP and ECT with bleomycin as an adjuvant therapeutic option for UM patients. The CAM assay represents a valuable and inexpensive in vivo platform that allows the rapid assessment of novel drug candidates, complementing in vitro experimentation prior to their in vivo application in animal models. The highly efficient engraftment of the patient-derived UM specimens onto the CAM enables the preservation of heterogeneity and pathophysiology of the original tumor, potentially reflecting the corresponding drug response. CaEP and ECT were extensively analyzed regarding their effect on proliferative activity, neovascular formations, melanocytic expansion, as well as on the induction of apoptosis and necrosis, which exhibit similar sensitivity following both EP-based approaches. These in vivo results from patient-derived samples underline the therapeutic potential of CaEP and ECT with bleomycin as effective adjuvant alternatives alongside established UM treatment regimens.

## Data Availability

The original contributions presented in the study are included in the article, further inquiries can be directed to the corresponding author.
